# Health-Related Quality of Life in Children With Congenital Hyperinsulinism

**DOI:** 10.3389/fendo.2019.00670

**Published:** 2019-10-01

**Authors:** Jonna M. E. Männistö, Jarmo Jääskeläinen, Hanna Huopio

**Affiliations:** ^1^Department of Pediatrics, Kuopio University Hospital and University of Eastern Finland, Kuopio, Finland; ^2^Department of Pediatrics, Kuopio University Hospital, Kuopio, Finland

**Keywords:** hypoglycemia, children and adolescent, KINDL-R®, persistent hyperinsulinemic hypoglycemia, transient hyperinsulinism

## Abstract

**Background:** Quality of life (QoL) has not been studied in patients with congenital hyperinsulinism (CHI).

**Objectives:** To examine whether the health-related quality of life (HRQoL) is worsened in patients with persistent or transient CHI.

**Methods:** We studied HRQoL of 65 children with CHI aged 3–17 years (60% males) recruited from the nationwide CHI registry. The median ages were 9.6 (range 3.5–16.3) and 7.4 (3.1–17.9) years in persistent (P-CHI, *n* = 33) and transient (T-CHI, *n* = 32) CHI groups, respectively. HRQoL was examined by generic KINDL-R questionnaire and the scores were compared to the age- and gender-specific reference values.

**Results:** In self-reports of subjects aged 11–17 years and in parent reports of children aged 3–17 years, P-CHI or T-CHI children did not have statistically lower scores in any of the six dimensions (physical well-being, emotional well-being, self-esteem, family, friends, and school) or in total scores compared to the reference values.

**Conclusions:** CHI is not associated with low HRQoL in childhood or adolescence.

## Introduction

Congenital hyperinsulinism (CHI) is a rare disease that manifests usually in neonates or young infants (1). It is characterized by inappropriate insulin secretion from pancreatic beta cells which leads to hypoglycemia. The disease holds a potential to affect quality of life (QoL) in many ways. Non-ketotic hypoglycemia induces a severe risk for brain damage and neurodevelopmental abnormalities are relatively common consequences of both persistent (P-CHI) and transient CHI (T-CHI) (2–4). The patients require daily oral or subcutaneous medication and some need to follow diet limitations. Focal disease is curable by localized resection of the pancreas, but a drug-resistant form of CHI may necessitate near-total pancreatectomy, which usually leads to insulin-dependent diabetes and in some cases to pancreatic exocrine dysfunction (1, 3).

Currently, there are no previous studies on QoL in CHI. In this study, we aimed to examine the health-related quality of life (HRQoL) in a nationwide cohort of Finnish patients with P-CHI or T-CHI.

## Materials and Methods

The participants were recruited from the nationwide CHI registry formed by diagnosis-based search from the hospital records of 19 largest Finnish hospitals. For parent reports, we sent the questionnaires to all the 149 families with 3–17-year-old patients (P-CHI, *n* = 57; T-CHI, *n* = 92). Of the respondents (*n* = 74), 65 were eligible for this study (P-CHI, *n* = 33; T-CHI, *n* = 32) after we excluded the patients having other diseases which may have an impact on QoL (atopic dermatitis, Beckwith-Wiedemann syndrome, Turner syndrome, cerebral palsy due to birth asphyxia or congenital intraventricular hemorrhage, and intellectual disability). Hence, 58% (33/57) and 35% (32/92) of the initially contacted parents in P-CHI and T-CHI groups (respectively) participated in this study. For self-reports, we sent the questionnaires to all the 43 patients who aged 11–17 years (P-CHI, *n* = 24; T-CHI, *n* = 19). Of the respondents (*n* = 20), 19 were eligible (P-CHI, *n* = 13; T-CHI, *n* = 6). Hence, 54% (13/24) and 32% (6/19) of the initially contacted patients in P-CHI and T-CHI groups (respectively) participated in this study.

The clinical data were collected from the hospital medical records. To ensure that we had real-time data of other diseases that potentially affect QoL, we also sent a self-made questionnaire considering current health, chronic diseases and requirement of extra developmental or educational support. The criteria for T-CHI were a neonatal onset (<28 days after birth) of hyperinsulinism and successful discontinuation of medication within 4 months.

HRQoL was measured using the age-specific Finnish versions of the generic Questionnaire for Measuring Health-related QoL in Children and Adolescents Revised Version (KINDL-R) (5–7). Since CHI manifests in early life, the KINDL-R encompassing children from the age of 3 years was the most appropriate. It consists of 24 items covering six dimensions: physical well-being, emotional well-being, self-esteem, family, friends, and school. Each item is rated on a 5-point Likert scale. The scores were transformed to values between 0 and 100, higher scores indicating better QoL. To examine statistical differences between the self- or parent report groups and the reference values, the scale scores were transformed to z-scores according to the age- and gender-specific reference values. One sample *t*-test was used to compare the z-scores to the validated German reference values of a healthy population sample (8, 9), as Finnish population norms were not available. The differences between participants and non-participants were analyzed with Mann-Whitney *U*-test and Fisher's exact test. The data were analyzed by IBM SPSS version 25.0 statistical software (SPSS Inc., Chicago, IL, USA). *P* < 0.05 was considered significant.

## Results

The clinical characteristics of the participants are presented in Table 1. Five patients with P-CHI were on medication and all of them had diffuse CHI according to their genetic diagnosis. All eight operated patients underwent a successful partial pancreas resection for focal CHI. Of the four patients having weekly symptoms of hypoglycemia, three were on medication and one was under consideration for medication.

**Table 1:** Clinical characteristic of the whole study population (*n* = 65).

	**Parent report group (*****n*** **=** **65)**		**Self-report group (*****n*** **=** **19)**	
	**P-CHI (*n* = 33)**	**T-CHI (*n* = 32)**	**P-CHI (*n* = 13)**	**T-CHI (*n* = 6)**
Male	58 (19)	63 (20)	46 (6)	50 (3)
Current age, years	9.6 (3.5–16.3)	7.4 (3.1–17.9)	14.5 (11.1–16.3)	12.3 (11.1–17.9)
Gestational age, weeks	38.9 (30.7–41.4)	37.4 (31.7–41.7)	39.1 (36.1–41.4)	36.7 (31.7–39.6)
Neonatal onset	60.6 (20)	NA	39 (5)	NA
Symptomatic at onset	57 (8)	57 (8)	77 (10)	33 (2)
Duration of medication, days	651 (35–3,783)	46 (6–112)	1130 (68–3,783)	52 (18–112)
Current medication	15 (5)	0 (0)	8 (1)	0 (0)
Operation	24 (8)	NA	23 (3)	NA
Current weekly symptoms of hypoglycaemia	12 (4)	0 (0)	0 (0)	0 (0)
CHI-associated gene variants detected	55 (18)	0 (0)	62 (8)	0 (0)
Medication for pancreatic exocrine dysfunction	6 (2)	0 (0)	8 (1)	0 (0)
Neurodevelopmental difficulties	15 (5)	13 (4)	31 (4)	17 (1)

*The data are presented as % (n) or median (range) and based on medical records and questionnaire*.

Five patients with P-CHI and four with T-CHI had mild neurodevelopmental difficulties referring to a reported diagnosis of pervasive or specific developmental disorder (ICD-10 codes F80-F89), or a need for educational or developmental (occupational, speech, or physiotherapy) support. None of the patients in this study had intellectual disability. Four P-CHI patients had significant long-term sequelae associated with CHI: hypoglycemic brain injury appearing as mild neurodevelopmental difficulties (*n* = 1), kidney dysfunction due to drug side effect (*n* = 1), and exocrine pancreatic dysfunction after surgery (*n* = 2).

**Figure 1:**
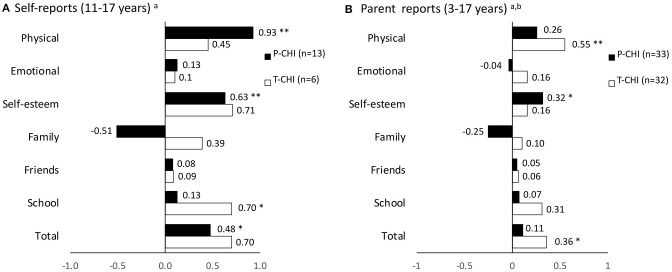
KINDL-R subscale and total mean z-scores according to age- and gender-specific reference values in the self-report groups **(A)** and parent report groups **(B)**. P-CHI, persistent CHI; T-CHI, transient CHI; *P < 0.05; **P < 0.001; ^a^reference values of BELLA study of the KiGGS (9); ^b^reference values of KiGGS study (8).

**Figure 2:**
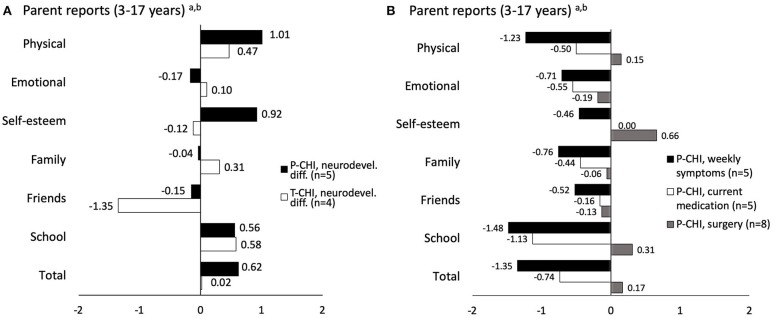
KINDL-R subscale and total mean z-scores according to age- and gender-specific reference values in the subgroups of children with mild neurodevelopmental difficulties **(A)**, current weekly symptoms, current medication, and surgical treatment **(B)**. P-CHI, persistent CHI; T-CHI, transient CHI; ^a^reference values of BELLA study of the KiGGS (9); ^b^reference values of KiGGS study (8).

The KINDL-R z-scores of the whole P-CHI and T-CHI groups are presented in Figure 1 and of the subgroups in Figure 2. In P-CHI group, the z-scores were statistically higher in the self-reports in physical well-being (*p* < 0.001), self-esteem (*p* = 0.002), and total scores (*p* = 0.038), as well as in self-esteem (*p* = 0.021) in parent reports. The z-scores of the T-CHI group were statistically higher in school-related well-being (*p* = 0.032) in self-reports, and in physical well-being (*p* < 0.001) and total scores (*p* = 0.013) in parent reports. Figure 1 indicates some, but not statistically significant difference in the dimension of family well-being between the P-CHI and T-CHI groups. Further, Figure 2 shows lower parent-reported scores (non-significant difference) of children with neurodevelopmental difficulties in the dimension of friends. Moreover, there were some, but not statistically significant differences in the parent-reported scores of children with weekly symptoms in the dimensions of physical well-being, school, and total scores, as well as in the parent-reported scores of children with current medication in the dimension of school (Figure 2). None of the dimensions showed statistically lower scores compared to the reference. The parent- and self-reported scale scores for the age groups are represented in Supplemental Tables 1, 2.

When the study participants were compared with nonparticipants in the self-report group, the participants with P-CHI were born later (39.1 vs. 37.0, *p* = 0.020) and had more often post-neonatal onset (>28 days after birth) (61% vs. 0%, *p* = 0.018). There were no other differences in birth size or weeks, age or symptoms at presentation, duration of medication, age, or in frequency of current medication, CHI-associated gene variants, epilepsy, or neurodevelopmental difficulties.

## Discussion

The current study is, to our knowledge, the first study to examine the HRQoL in patients with CHI. In this population-based cohort, self-, and parent reports of children with P-CHI or T-CHI did not show statistically lower mean scores in any of the six dimensions and total scores compared to the reference values of the HRQoL questionnaire.

Previous studies on QoL in severe pediatric diseases have shown mainly impaired, but also contradictory results. Lower QoL scores than the reference have generally been found in, e.g., asthma, type 1 diabetes, epilepsy, eczema, and active juvenile arthritis (10–13). However, in some chronic and severe diseases QoL has been found to be equally good or even better than in healthy controls or the reference, and longitudinal studies on, for example juvenile arthritis, epilepsy, and immune thrombocytopenia have reported improved QoL with a longer duration of the disease, reaching the level of healthy peers (14–16). Moreover, in a study on congenital adrenal hyperplasia, QoL was even better than the reference (17).

In the current study, CHI was not associated with worsened HRQoL in childhood and adolescence. There are several aspects that may contribute to these findings. First, in previous studies, decreased disease activity, resilience, and adaptation to the disease over time associated with favorable results (15, 18, 19). Achieving stable asymptomatic normoglycemia in CHI may be challenging in some patients, but eventually, most patients respond to medical or surgical treatment over time. It is to notice that the majority of the patients in our study had been able to discontinue medication, whereas the subgroups of patients with weekly symptoms or current medication (i.e., active disease) showed some tendency for lower scores in some HRQoL dimensions. Second, education about the disease and its management, skill training, as well as nutritional advice may improve perceived QoL, as reported in a previous meta-analysis (18). Finally, generic instruments may underestimate disease-specific impairment in HRQoL (16, 20, 21). For example, a review of HRQoL studies in type 1 diabetes showed a similar generic QoL compared to healthy controls despite of disease-specific QoL problems (20). There are no disease-specific questionnaires in CHI, but in our study, most families did not even respond to the disease-subscale questionnaire, further indicating a minor impact of the disease on their well-being.

In previous studies, children with several chronic conditions have generally demonstrated lower HRQoL compared to those with only one disease (12). In our cohort, four patients with P-CHI had long-term sequelae of hypoglycemia (in most patients, mild neurodevelopmental difficulties could not be retrospectively associated with CHI). These patients did not have substantially lower QoL scores compared to the reference. This, again, may be due to the coping strategies and additional support to the families, as discussed. Our results also reflect improved management of these recently born and treated patients. No patient in our study was treated with subtotal pancreatectomy associated with a high risk for insulin-dependent diabetes and pancreatic exocrine dysfunction. Moreover, none of the patients had intellectual disability due to a severe hypoglycemic insult.

The weakness of this study is the rather low participation rates and numbers, which may reflect the large number of already recovered participants and also the milder nature of T-CHI. The strengths of this study were the population-based setting, exclusion of other conditions with a potential impact on HRQoL, and comparison to age- and gender-specific reference values. Furthermore, the participants did not significantly differ from the non-participants in clinical characteristics. KINDL-R is proved to be a reliable and valid instrument in healthy and sick children, and it has been widely used internationally (22–25). Further, in Finland, the Finnish TOIMIA (National Expert Network on Measurement of Functioning) has stated it well-applicable (26).

We conclude that the HRQoL in patients with P-CHI or T-CHI who have been treated mainly in the twenty-first century is not impaired in later childhood or adolescence. Despite, the fact that our study cohort represents only a small number of patients these results are encouraging and highlight the significance of the modern individual treatment strategies of CHI.

## Data Availability Statement

All datasets generated for this study are included in the manuscript/Supplementary Files.

## Ethics Statement

The studies involving human participants were reviewed and approved by Ethics Committee of the Northern Savo Hospital District. Written informed consent to participate in this study was provided by the participants' legal guardian/next of kin.

## Author Contributions

JM collected the data, performed the statistical analysis, and wrote the first draft of the manuscript. All authors contributed to conception and design of the study, manuscript revision, read and approved the submitted version.

### Conflict of Interest

The authors declare that the research was conducted in the absence of any commercial or financial relationships that could be construed as a potential conflict of interest.

## References

[1] Galcheva S Al-Khawaga S Hussain K. Diagnosis and management of hyperinsulinaemic hypoglycaemia. Best Pract Res Clin Endocrinol Metab. (2018) 32:551–73. 10.1016/j.beem.2018.05.014 30086874

[2] Avatapalle HB Banerjee I Shah S Pryce M Nicholson J Rigby L. Abnormal neurodevelopmental outcomes are common in children with transient congenital hyperinsulinism. Front Endocrinol. (2013) 4:60. 10.3389/fendo.2013.00060 23730298 PMC3657691

[3] Lord K Radcliffe J Gallagher PR Adzick NS Stanley CA De Leon DD. High risk of diabetes and neurobehavioral deficits in individuals with surgically treated hyperinsulinism. J Clin Endocrinol Metab. (2015) 100:4133–9. 10.1210/jc.2015-2539 26327482 PMC4702456

[4] Ludwig A Enke S Heindorf J Empting S Meissner T Mohnike K. Formal neurocognitive testing in 60 patients with congenital hyperinsulinism. Horm Res Paediatr. (2018) 89:1–6. 10.1159/000481774 29151084

[5] Ravens-Sieberer U Bullinger M. Assessing health-related quality of life in chronically ill children with the German KINDL: first psychometric and content analytical results. Qual Life Res. (1998) 7:399–407. 10.1023/A:1008853819715 9691720

[6] Ravens-Sieberer U Bullinger M News from the KINDL-questionnaire - a new version for adolescents. Qual Life Res. (1998) 7:653. 10.1023/a:1008853819715 9691720

[7] The KINDL(R) Homepage Available from: https://www.kindl.org/english/questionnaires/

[8] Ravens-Sieberer U Ellert U Erhart M. Health-related quality of life of children and adolescents in Germany. Norm data from the German Health Interview and Examination Survey (KiGGS). Bundesgesundheitsblatt Gesundheitsforschung Gesundheitsschutz. (2007) 50:810–8. 10.1007/s00103-007-0244-4 17514467

[9] Ravens-Sieberer U Erhart M Wille N Bullinger M BELLA study group. Health-related quality of life in children and adolescents in Germany: results of the BELLA study. Eur Child Adolesc Psychiatry. (2008) 17 (Suppl 1):148–56. 10.1007/s00787-008-1016-x 19132314

[10] Sawyer MG Reynolds KE Couper JJ French DJ Kennedy D Martin J. Health-related quality of life of children and adolescents with chronic illness–a two year prospective study. Qual Life Res. (2004) 13:1309–19. 10.1023/B:QURE.0000037489.41344.b2 15473509

[11] Varni JW Limbers CA Burwinkle TM. Impaired health-related quality of life in children and adolescents with chronic conditions: a comparative analysis of 10 disease clusters and 33 disease categories/severities utilizing the PedsQL 4.0 Generic Core Scales. Health Qual Life Outcomes. (2007) 5:43–7525. 10.1186/1477-7525-5-43 17634123 PMC1964786

[12] Bai G Herten MH Landgraf JM Korfage IJ Raat H. Childhood chronic conditions and health-related quality of life: findings from a large population-based study. PLoS ONE. (2017) 12:e0178539. 10.1371/journal.pone.0178539 28575026 PMC5456082

[13] Silva N Pereira M Otto C Ravens-Sieberer U Canavarro MC Bullinger M. Do 8- to 18-year-old children/adolescents with chronic physical health conditions have worse health-related quality of life than their healthy peers? A meta-analysis of studies using the KIDSCREEN questionnaires. Qual Life Res. (2019) 28:1725–50. 10.1007/s11136-019-02189-7 31055778

[14] Speechley KN Ferro MA Camfield CS Huang W Levin SD Smith ML. Quality of life in children with new-onset epilepsy: a 2-year prospective cohort study. Neurology. (2012) 79:1548–55. 10.1212/WNL.0b013e31826e25aa 23019268 PMC3475627

[15] Listing M Monkemoller K Liedmann I Niewerth M Sengler C Listing J. The majority of patients with newly diagnosed juvenile idiopathic arthritis achieve a health-related quality of life that is similar to that of healthy peers: results of the German multicenter inception cohort (ICON). Arthritis Res Ther. (2018) 20:106–18. 10.1186/s13075-018-1588-x 29848349 PMC5977761

[16] Trotter P Hill QA. Immune thrombocytopenia: improving quality of life and patient outcomes. Patient Relat Outcome Meas. (2018) 9:369–84. 10.2147/PROM.S140932 30568522 PMC6267629

[17] Jaaskelainen Voutilainen R. Long-term outcome of classical 21-hydroxylase deficiency: diagnosis, complications and quality of life. Acta Paediatr. (2000) 89:183–7. 10.1111/j.1651-2227.2000.tb01213.x 10709888

[18] Kim M Kim K Kim JS. Impact of resilience on the health-related quality of life of adolescents with a chronic health problem: a structural equation approach: resilience and health-related quality of life of adolescents. J Adv Nurs. (2019) 75:801–11. 10.1111/jan.13888 30375047

[19] Otto C Barthel D Klasen F Nolte S Rose M Meyrose AK. Predictors of self-reported health-related quality of life according to the EQ-5D-Y in chronically ill children and adolescents with asthma, diabetes, and juvenile arthritis: longitudinal results. Qual Life Res. (2018) 27:879–90. 10.1007/s11136-017-1753-8 29189988

[20] Nieuwesteeg A Pouwer F van der Kamp R van Bakel H Aanstoot HJ Hartman E. Quality of life of children with type 1 diabetes: a systematic review. Curr Diabetes Rev. (2012) 8:434–43. 10.2174/157339912803529850 22934548

[21] Hukkinen M Merras-Salmio L Pakarinen MP. Health-related quality of life and neurodevelopmental outcomes among children with intestinal failure. Semin Pediatr Surg. (2018) 27:273–9. 10.1053/j.sempedsurg.2018.07.004 30342603

[22] Solans M Pane S Estrada MD Serra-Sutton V Berra S Herdman M. Health-related quality of life measurement in children and adolescents: a systematic review of generic and disease-specific instruments. Value Health. (2008) 11:742–64. 10.1111/j.1524-4733.2007.00293.x 18179668

[23] Kojima N Ohya Y Futamura M Akashi M Odajima H Adachi Y. Exercise-induced asthma is associated with impaired quality of life among children with asthma in Japan. Allergol Int. (2009) 58:187–92. 10.2332/allergolint.08-OA-0034 19240375

[24] Freeman AJ Youngstrom EA Michalak E Siegel R Meyers OI Findling RL. Quality of life in pediatric bipolar disorder. Pediatrics. (2009) 123:e446–52. 10.1542/peds.2008-0841 19254981

[25] Stahl-Pehe A Strassburger K Castillo K Bachle C Holl RW Lange K. Quality of life in intensively treated youths with early-onset type 1 diabetes: a population-based survey. Pediatr Diabetes. (2014) 15:436–43. 10.1111/pedi.12096 25298998

[26] Finnish National Institute for Health and Welfare TOIMIA Functioning Measures Database. Available online at: https://thl.fi/en/web/functioning/toimia-functioning-measures-database.

