# Supplementary material for: Health-Related Quality of Life in Children With Congenital Hyperinsulinism
Source: Front Endocrinol (Lausanne). 2019 Oct 1;10:670. doi: 10.3389/fendo.2019.00670 (PMC6779698; doi:10.3389/fendo.2019.00670)
Supplement: Supplementary file 1 [file Table_1.pdf]

Supplemental Table 1. Parent-reported quality of life for 3-17-year-old patients in the persistent (P-CHI) or transient (T-CHI) CHI groups (n=65).

Scale scores of KINDL-R [a]		*N*	Physical	Emotional	Self-esteem	Family	Friends	School	Total
			mean (SD)	mean (SD)	mean (SD)	mean (SD)	mean (SD)	mean (SD)	mean (SD)
3 to 6 yr	P-CHI	12	71.9 (21.7)	80.7 (12.1)	76.6 (11.0)	72.9 (10.4)	78.1 (9.4)	76.4 (15.0)	76.1 (10.4)
	T-CHI	12	89.2 (9.1)	83.9 (10.1)	77.6 (11.4)	77.6 (11.4)	81.8 (9.8)	80.7 (12.3)	81.3 (6.3)
	Reference [b]		80.2 (15.7)	83.0 (11.4)	80.7 (11.9)	80.7 (11.9)	79.7 (12.3)	83.8 (12.5)	80.0 (8.1)
7 to 10 yr	P-CHI	6	85.4 (5.1)	80.2 (17.4)	70.8 (3.2)	83.3 (7.6)	80.2 (10.8)	88.5 (9.2)	81.4 (6.9)
	T-CHI	13	87.0 (13.2)	82.7 (10.9)	75.0 (6.8)	80.8 (11.3)	77.9 (15.0)	90.4 (6.6)	82.3 (7.3)
	Reference [c]		80.6 (16.3)	82.7 (12.1)	71.4 (13.2)	79.9 (12.6)	78.5 (13.0)	83.1 (14.2)	79.4 (9.1)
11 to 13 yr	P-CHI	7	91.1 (8.0)	83.9 (8.0)	72.3 (3.3)	75.0 (10.8)	78.6 (14.4)	83.9 (8.7)	80.8 (4.1)
	T-CHI	6	87.5 (9.7)	88.5 (4.7)	66.7 (10.2)	86.5 (7.3)	79.2 (11.6)	89.6 (7.6)	83.0 (5.5)
	Reference [c]		76.1 (15.8)	79.8 (12.8)	68.1 (13.8)	76.7 (14.6)	77.1 (13.4)	76.8 (14.8)	75.7 (9.9)
14 to 17 yr	P-CHI	8	87.5 (10.6)	79.7 (11.9)	78.1 (15.7)	75.0 (18.3)	80.5 (12.2)	75.0 (17.0)	79.3 (10.9)
	T-CHI	1	93.8 (0.0)	75.0 (0.0)	75.0 (0.0)	93.8 (0.0)	75.0 (0.0)	75.0 (0.0)	81.3 (0.0)
	Reference [c]		73.3 (18.3)	79.8 (13.1)	67.2 (15.0)	76.6 (15.2)	78.2 (13.6)	69.6 (15.7)	74.1 (10.4)

P-CHI, persistent CHI; T-CHI, transient CHI; [a] transformed scale scores ranging from 0 to 100, higher scores indicating better quality of life; [b] KiGGS study (Ravens-Sieberer et al., 2007); [c] BELLA study of the KiGGS (Ravens-Sieberer et al., 2008).

Supplemental Table 2. Self-reported quality of life for 7-17-year-old patients in the persistent (P-CHI) or transient (T-CHI) CHI groups (n=19).

Scale scores ofr KINDL-R®		*N*	Physical	Emotional	Self-esteem	Family	Friends	School	Total
			mean (SD)	mean (SD)	mean (SD)	mean (SD)	mean (SD)	mean (SD)	mean (SD)
11 to 13 yr	P-CHI	5	87.5 (7.7)	85.0 (7.1)	71.3 (3.4)	71.3 (9.5)	78.8 (10.5)	85.0 (9.5)	79.8 (4.7)
	T-CHI	5	78.8 (24.0)	85.0 (5.6)	70.0 (17.3)	87.5 (9.9)	82.5 (8.1)	82.5 (10.3)	81.0 (9.1)
	Reference [b]		76.1 (15.8)	79.8 (12.8)	68.1 (13.8)	76.7 (14.6)	77.1 (13.4)	76.8 (14.8)	75.7 (9.9)
14 to 17 yr	P-CHI	8	83.6 (10.5)	82.0 (7.0)	68.8 (13.8)	77.3 (17.3)	85.2 (10.5)	59.4 (16.4)	76.0 (9.4)
	T-CHI	1	81.3 (0.0)	75.0 (0.0)	68.8 (0.0)	100.0 (0.0)	81.3 (0.0)	81.3 (0.0)	81.3 (0.0)
	Reference [b]		73.3 (18.3)	79.8 (13.1)	67.2 (15.0)	76.6 (15.2)	78.2 (13.6)	69.6 (15.7)	74.1 (10.4)

P-CHI, persistent CHI; T-CHI, transient CHI; [a] transformed scale scores ranging from 0 to 100, higher scores indicating better quality of life; [b] BELLA study of the KiGGS (Ravens-Sieberer et al., 2008).
